# Para Quais Pacientes Infectados pelo HIV a Aspirina e as Estatinas São Boas?

**DOI:** 10.36660/abc.20210560

**Published:** 2021-08-09

**Affiliations:** 1 Universidade Johns Hopkins Departamento de Medicina Divisão de Cardiologia Baltimore USA Divisão de Cardiologia, Departamento de Medicina, Universidade Johns Hopkins y, Baltimore - USA; 2 Universidade de Paris Departamento de Cardiologia Hospital Lariboisiere Paris França Departamento de Cardiologia, Universidade de Paris, Hospital Lariboisiere, Inserm, UMRS 942, Paris - França; 3 Universidade Estadual de Campinas Faculdade de Ciências Médicas São Paulo Brasil Disciplina de Cardiologia, Faculdade de Ciências Médicas– Universidade Estadual de Campinas (UNICAMP), Campinas, São Paulo - Brasil

**Keywords:** HIV, HIV/infecção, Anti Agentes HIV/uso terapêutico, Doenças Cardiovasculares/complicações, Mortalidade, Fatores de Risco, Aspirina, Estatinas, Aterosclerose, Endotélio

Embora os avanços no tratamento antirretroviral revolucionaram o prognóstico dos pacientes infectados pelo vírus da imunodeficiência humana (HIV), as complicações cardiovasculares continuam a principal causa de morte nesses pacientes, principalmente devido a um aumento no risco cardiovascular em comparação à população geral.^1^ Programas de prevenção a doenças cardiovasculares têm enfatizado a importância do controle de fatores de risco tradicionais nas estratégias de avaliação de risco. No entanto, indivíduos infectados pelo HIV têm um risco cardiovascular residual para eventos cardiovasculares que pode justificar um tratamento preventivo adicional. De fato, em comparação a indivíduos não infectados pelo HIV, os níveis de inflamação são mais altos em pacientes infectados pelo HIV, mesmo aqueles com controle viral, e essa inflamação é um fator importante na gênese da aterosclerose.^2^ Assim, estratégias de prevenção a doenças cardiovasculares com foco em pacientes com infecção pelo HIV são necessárias.^3^ No arsenal terapêutico da prevenção cardiovascular, a aspirina e as estatinas são os pilares do manejo de pacientes infectados por HIV. Ambos os medicamentos possuem efeitos pleiotrópicos, incluindo efeitos imunomodulatórios, antiplaquetários, e anti-inflamatórios que melhoram a função endotelial e previnem a progressão do espessamento da carótida desses pacientes.^4^ Contudo, a prescrição de aspirina e estatinas entre pacientes infectados por HIV continua aquém do desejado, já que somente 50% dos pacientes são tratados adequadamente. Apesar de vários estudos terem investigado os efeitos de estatinas e da aspirina na redução da inflamação, os resultados desses estudos são contraditórios.^5^^,^^6^

Nesta edição dos Arquivos Brasileiros de Cardiologia, Santos Jr. et al.^7^ relatam o efeito do uso de uma combinação de atorvastatina e aspirina por seis meses quanto à melhoria da função endotelial e ao espessamento da carótida em uma coorte de 38 pacientes com carga viral controlada. A melhora na função endotelial foi avaliada pelo método de dilatação mediada por fluxo. Os autores mostraram uma relação entre a resposta ao tratamento e idade; uma maior resposta foi observada em indivíduos com idade maior a 40 anos. Esse resultado pode ser explicado pelo fato de que, provavelmente, indivíduos mais velhos têm uma maior exposição à inflamação causada pelo HIV. Vários estudos já demonstraram que esses mesmos pacientes também possuem um risco cardiovascular mais elevado devido à inflamação crônica. Portanto, este estudo corrobora a prescrição de uma combinação de atorvastatina e aspirina para a prevenção primária de eventos cardiovasculares em pacientes infectados pelo HIV, particularmente para aqueles com idade maior que 40 anos de idade. Além disso, alguns dos achados desse estudo sugerem que as mulheres soropositivas para o HIV podem apresentar uma melhor resposta a essa combinação de medicamentos que os homens. Considerando que a terapia tripla atualmente utilizada tem um efeito significativo sobre a inflamação, um mecanismo intrinsicamente ligado à progressão da aterosclerose poderia explicar a maior reposta em mulheres que em homens.

O estudo de Santos Jr. et al.,^7^ serve como base para entender os fatores que influenciam na melhoria da função endotelial em pacientes infectados pelo HIV recebendo atorvastatina e aspirina. Desses fatores, a idade mais avançada parece ser um dos mais importantes. Os resultados são motivadores, ao sugerirem que a combinação de aspirina e estatinas reduz efetivamente ou mesmo reverte alguns dos efeitos deletérios induzidos pelo HIV. Estudos similares envolvendo um grande número de indivíduos são necessários para confirmar a hipótese dos autores e corroborar o uso precoce da combinação de atorvastatina com aspirina em pacientes infectados pelo HIV acima de 40 anos de idade, mesmo naqueles em baixo risco cardiovascular, para a prevenção de doença cardiovascular. Este estudo fornece contribuição para a evidência clínica dos efeitos positivos de aspirina e estatinas, usadas em combinação com terapia antiretroviral em paciente com HIV, após devida consideração das possíveis interações medicamentosas. Os resultados apresentados por Santos Jr et al.,^7^ apresentam uma base fascinante para essas considerações; no entanto, é necessário destacar algumas limitações importantes do estudo. Primeiramente, o estudo não foi um ensaio clínico randomizado, e a exposição a estatinas foi relativamente curta em comparação a outros estudos. Além disso, o estudo incluiu uma coorte de pacientes com HIV com um perfil de baixo risco cardiovascula, e baixo grau de inflamação, confirmado pelos baixos níveis de marcadores inflamatórios. Assim, é importante considerar que o impacto de aspirina e estatinas sobre a remodelação vascular de pacientes infectados pelo HIV com esse perfil clínico pode não ser relevante.
