# Supplementary material for: Para Quais Pacientes Infectados pelo HIV a Aspirina e as Estatinas São Boas?
Source: Arq Bras Cardiol. 2021 Aug 9;117(2):376–7. [Article in Portuguese] doi: 10.36660/abc.20210560 (PMC8395806; doi:10.36660/abc.20210560)
Supplement: Translation

# Translation: For which HIV Patients Aspirin and Statins are Good?

**Affiliations:** 1 Baltimore USA Divisão de Cardiologia, Departamento de Medicina, Universidade Johns Hopkins y, Baltimore - USA; 2 Paris France Departamento de Cardiologia, Universidade de Paris, Hospital Lariboisiere, Inserm, UMRS 942, Paris - France; 3 São Paulo Brazil Disciplina de Cardiologia, Faculdade de Ciências Médicas– Universidade Estadual de Campinas (UNICAMP), Campinas, São Paulo - Brazil

**Keywords:** HIV, HIV/infection, Anti-HIV Agents/therapeutic use, Cardiovascular Diseases/complications, Mortality; Risk Factors, Aspirin, Statins, Atherosclerosis, Endothelium

While advances in antiretroviral treatment have revolutionized the prognosis of human immunodeficiency (HIV)-infected patients, cardiovascular complications remain the leading cause of death in these patients mainly due to an increased cardiovascular risk compared to the general population.^1^ Cardiovascular prevention programs have highlighted the importance of controlling traditional risk factors in risk evaluation strategies. However, HIV-infected individuals at low cardiovascular risk have a considerable residual cardiovascular risk for events that may justify additional preventive treatment. Indeed, compared with non-HIV-infected individuals, inflammation levels are higher in HIV-infected patients, even those with viral control, and this inflammation is an important factor in the genesis of atherosclerosis.^2^ Therefore, effective cardiovascular prevention strategies targeting HIV population are needed.^3^ In the therapeutic arsenal of cardiovascular prevention, aspirin and statins are the cornerstones of the management of HIV-infected patients. Both drugs have pleiotropic effects, including immunomodulatory, anti-thrombogenic, and anti-inflammatory effects, that improve endothelial function and prevent the progression of carotid thickening in these patients. However, the prescription of aspirin and statins in HIV-infected patients remains largely suboptimal, with only 50% of patients adequately treated.^4^ Although several studies have investigated the effects of statins and aspirin in decreasing inflammation, the results of these studies are contradictory.^5^^,^^6^

In this issue of the *Arquivos Brasileiros de Cardiologia*, Santos Jr et al.^7^ report the effect of the use of a combination of atorvastatin and aspirin for six months regarding the endothelial function improvement and carotid thickness in a cohort of 38 patients with HIV infection with viral control. Improvement in endothelial function was assessed using the brachial artery flow-mediated dilation. The authors have shown a relationship between treatment response and age; a stronger response was observed in individuals older than 40 years. This result may be explained by the fact that probably older individuals had a longer exposure to inflammation caused by HIV. Several studies have shown that these same patients also have a higher cardiovascular risk due to chronic inflammation. Therefore, this study supports the prescription of a combination of atorvastatin and aspirin for the primary prevention of cardiovascular events in HIV-infected patients, particularly for those over 40 years of age. In addition, some of the findings of this study suggest that HIV-positive women may have a better response to this drug combination than men. Considering that the currently used triple therapy has a significant effect on inflammation, a mechanism intrinsically linked to the progression of atherosclerosis could explain the greater response in women than in men.

The work by Santos Jr et al.^7^ is a basis for understanding the factors influencing the improvement of endothelial function in HIV-infected patients receiving atorvastatin and aspirin. Of these factors, older age appears to be one of the most important. Encouragingly, the results suggest that the combination of aspirin and statins effectively reduces or even reverses some of the deleterious effects induced by HIV. Similar studies involving a larger number of individuals are needed to confirm the authors’ hypothesis and to the early use of the combination of atorvastatin and aspirin in HIV-infected patients over 40 years of age, even in those at low cardiovascular risk, for the prevention of cardiovascular disease. This study adds to the clinical evidence on positive effects of aspirin and statins in combination with antiretroviral therapy in HIV patients, after due consideration of possible drug interactions. The results presented by Santos Jr et al.^7^ provide a fascinating basis for these considerations; however, it is essential to highlight some important limitations of the study. First, the study was not a randomized clinical trial and the exposure to statins was relatively short compared to other studies. In addition, it included a cohort of patients with HIV with a low cardiovascular risk profile and low inflammation as confirmed by the low levels of inflammatory markers. Importantly, the impact of aspirin and statins on vascular remodeling of HIV patients with this clinical profile may not be relevant.
